# Clinician-documented Firearm Access and Safety Interventions for Veterans Receiving Suicide Risk Evaluation in VA Emergency Care Settings

**DOI:** 10.5811/westjem.50852

**Published:** 2026-05-19

**Authors:** Joseph A Simonetti, Samuel E King, Ryan Holliday, Gabriela K Khazanov, Alexandra Smith, Nazanin Bahraini, Lisa A Brenner, Bridget B. Matarazzo

**Affiliations:** * Veterans Health Administration, Rocky Mountain Regional VA Medical Center, Department of Rocky Mountain Mental Illness Research, Education and Clinical Center for Suicide Prevention, Aurora, Colorado; † University of Colorado Anschutz School of Medicine, Firearm Injury Prevention Initiative, Aurora, Colorado; ‡ University of Colorado Anschutz School of Medicine, Department of Physical Medicine and Rehabilitation, Aurora, Colorado; § University of Colorado Anschutz School of Medicine, Department of Neurology, Aurora, Colorado; || University of Colorado Anschutz School of Medicine, Department of Psychiatry, Aurora, Colorado; # Ferkauf Graduate School of Psychology, Yeshiva University, Bronx, New York; ** Veterans Health Administration, Rocky Mountain Regional VA Medical Center, Brain Health Coordinating Center, Aurora, Colorado

## Abstract

**Background:**

The success of clinical programs aimed at preventing suicide risk depends in part on whether they can be used to identify and act upon risk factors for suicide. Our aim in this study was to describe frequency of clinician documentation of firearm access and the delivery of safety interventions among patients who received a suicide risk evaluation in Veterans Health Administration (VHA) emergency departments (ED) or urgent care (UC) settings.

**Methods:**

We used electronic health record data of patients who received care in VHA ED/UC settings January 2021–October 2022 and underwent suicide risk evaluation by clinicians using the Veterans Affairs (VA) Comprehensive Suicide Risk Evaluation (CSRE) prior to discharging home. The proportion of patients with self-reported firearm access was identified from clinician-documented CSRE templates. Among those who reported firearm access, we identified the proportion who received any safety intervention (delivery of lethal means safety counseling and/or distribution of firearm cable locks per CSRE documentation, or update/creation/review of a VA Safety Plan) within 24 hours of the ED/UC encounter. We compared differences using chi-square or Fisher exact tests for categorical outcomes and analysis of variance or independent sample *t*-tests for continuous outcomes.

**Results:**

Of 17,194 patients who were discharged home, 15.2% were documented as having firearm access (8.5% access to “other” lethal means, 68.8% no lethal means access, 7.4% unknown access). Of 2,624 patients with documented firearm access, 80.6% were documented as having received a safety intervention. Of those, 56.8% received lethal means safety counseling, 13.2% received a firearm cable lock, and 88.6% reviewed or completed a new or updated VA Safety Plan.

**Conclusion:**

Among patients who underwent suicide risk evaluation prior to discharging home from a Veterans Health Administration ED/UC setting, a low percentage were documented as having firearm access. Of those with firearm access, a large majority received at least one safety intervention. System-wide strategies to encourage delivery of safety interventions can reach a large proportion of at-risk patients. Additional efforts are needed to increase reporting and documentation of firearm access.

## INTRODUCTION

The age-adjusted suicide rate in the United States (U.S.) increased by approximately 30% from 2001 to 2021, and more than 48,000 individuals died from suicide in 2021.^1^ In response to this, a variety of interventions aimed at identifying and mitigating suicide risk have been deployed in healthcare systems.^2^–^4^ A common limitation of these efforts is that interventions aimed at identifying and addressing suicide risk have typically been centered within mental health settings, thereby focusing on individuals with known mental health conditions. This approach is insufficient in identifying all patients at risk for suicidal behavior.^5^–^7^ Nearly one-half of individuals who die by suicide in the U.S. are not diagnosed with a mental health condition prior to their death.^8^,^9^ Further, there are other important risk factors for suicide, such as firearm access and physical health conditions (eg, cancer), and suicide decedents commonly seek care in non-mental health settings prior to their deaths.^10^–^12^

One approach to address these challenges is to expand the implementation of suicide risk screening and evaluation to other clinical settings and patient groups. In 2018, the Veterans Health Administration (VHA) implemented a nationwide effort to expand suicide risk screening and evaluation among its patient population, known as Veterans Affairs Suicide Risk Identification Strategy (VA Risk ID).^13^,^14^ In addition to standardizing procedures for risk screening and evaluation, VA Risk ID included the extension of processes to non-mental health settings, such as specialty medical and emergency care settings. Early evaluations of this initiative have demonstrated that identification of suicide risk through VA Risk ID is associated with increased mental health follow-up and engagement, particularly for patients who are not already connected to mental health care.^15^

The effectiveness of VA Risk ID and similar efforts in other healthcare systems will depend in part on whether the clinical processes can be used to identify actionable risk factors for suicide and subsequent implementation of evidence-informed interventions. Firearm access is an independent risk factor for suicide and is common among veterans; in 2022, 73% of veteran suicides were attributed to firearm injury.^11^,^16^,^17^ Evaluation procedures used in VA Risk ID include assessment of firearm access among care-seeking veterans and documentation of safety interventions indicated for those who endorse firearm access. Our goal in this study was to describe the frequency of clinician-documented firearm access among veterans who sought care in VHA emergency department (ED)/urgent care (UC) settings and received suicide risk evaluation. We further determined which, if any, safety interventions (eg, lethal means counseling) were provided to patients who endorsed firearm access.

## METHODS

We conducted a nationwide, retrospective study of electronic health records of patients who received care in VHA ED or UC settings. This study was determined to be quality improvement and, thus, review by the institutional review board was not required.

**Population Health Research Capsule**

What do we already know about this issue?

*The Veterans Health Administration (VHA) has incorporated screening for firearm access for patients with elevated suicide risk*.

What was the research question?

*Of patients with elevated suicide risk discharged from emergency departments, what proportions have firearm access and receive firearm-related interventions?*

What was the major finding of the study?

*Of 17,194 discharged patients, 15.2% had firearm access and 80.6% of those patients received a safety intervention*.

How does this improve population health?

*System-wide strategies to encourage delivery of safety interventions can reach a large proportion of at-risk patients*.

### Veterans Affairs Suicide Risk Identification Strategy in Emergency Department/Urgent Care Settings

Per VA Risk ID, all patients accessing VHA ED/UC services are mandated to be screened and/or evaluated for suicide risk. Screening is conducted using the Columbia Suicide Severity Rating Scale Screener (C-SSRS),^18^ which assesses past month suicidal ideation, including method, intent, plan, and both lifetime and recent (prior three month) suicidal behavior. A positive C-SSRS screen is defined as a “yes” response regarding suicide method, intent, plan, and/or recent suicidal behavior. Clinicians are required to complete the VA Comprehensive Suicide Risk Evaluation (CSRE) and document results within 24 hours for patients who screen positive.^14^ The CSRE is a VHA-specific, templated clinical tool that facilitates the collection of patient-reported data on suicide risk factors, including firearm access, and protective factors that are then used to inform a determination of acute (low, intermediate, high) and chronic (low, intermediate, high) suicide risk. Based on this evaluation and stratification, clinicians then document evidence-informed suicide risk interventions (eg, lethal means counseling; safety planning; naloxone distribution; outpatient referral; hospitalization) that will best meet the patient’s needs and preferences. In some circumstances (eg, patient self-disclosure of recent suicide attempt during intake), clinicians may forgo C-SSRS screening and administer the CSRE. Additional information on VA Risk ID and the CSRE have been published previously.^14^

Veterans Hospital Administration policy also requires that patients who are discharged home and stratified at intermediate (acute or chronic) or high (acute or chronic) suicide risk in ED/UC settings receive a new or updated VA Safety Plan (or review an existing one), which must be documented within 24 hours of their ED discharge. The VA Safety Plan is a brief, structured intervention designed to mitigate future risk by providing individuals with a written, personalized plan to be used before the onset or during a suicidal crisis.^19^ It is developed collaboratively between a clinician and a patient and has six main steps. Step 6 is focused on identifying strategies for “making one’s environment safer” and includes elements of lethal means safety, such as use of secure firearm storage practices.

### Setting, Patients, and Data Source

We included all patients who sought care in VHA ED/UC settings from January 2021–October 2022, received a CSRE that was documented from one hour prior to ED/UC arrival until one hour after ED/UC discharge, and whose discharge disposition was “home.” All data were abstracted from the VA Corporate Data Warehouse.

### Outcomes and Variables

The primary outcomes of this study were 1) patient-reported, clinician-documented firearm access, and 2) clinician-documented delivery of safety interventions during the ED/UC encounter. Firearm access was abstracted from CSREs conducted during the ED/UC encounter and assessed using the item “Does the veteran have access to lethal means?” Response options include “yes,” “no,” and “unknown,” and clinicians are required to respond specifically to prompts about “firearms” and “other lethal means.”

Delivery of safety interventions was abstracted from templated content in the CSRE Risk Mitigation Plan, in which clinicians document which prevention strategies are indicated. Clinicians have the option to document delivery of “lethal means safety counseling,” which may also include the provision of no-cost firearm cable locks, and the review of or completion of a new or updated VA Safety Plan. We considered a patient to have received a safety intervention if the clinician documented delivery of “lethal means safety counseling” (with or without distribution of a firearm cable lock) in the CSRE or review or creation of a new or updated safety plan (documented in CSRE Risk Mitigation Plan or in a separate standardized note template). We abstracted safety plan data from notes entered 24 hours prior to the clinical encounter (because repeating a VA Safety Plan in the same 24-hour period is not necessarily clinically warranted) to 24 hours after discharge.

To characterize the study population, we also abstracted other variables including the following: age; sex; race; ethnicity; marital status; the presence of mental health diagnoses; prior engagement with homeless services; C-SSRS Screener results documented from one hour prior to the index encounter until the end of that calendar day; and CSRE risk stratification (acute: low, intermediate, high; chronic: low, intermediate, high).

### Data Analysis

We began by categorizing the study population into four mutually exclusive groups, including those with firearm access, access to lethal means but not firearms, access to no lethal means, and those with unknown access to lethal means. We then described the demographic, clinical, and suicide risk-stratification characteristics of the study population and compared differences across groups. Among those with patient-reported, clinician-documented firearm access, we quantified the proportion of patients for whom a clinician documented the delivery of a safety intervention (lethal means safety counseling (with or without distribution of a cable lock] and/or review or creation of a new or updated VA Safety Plan). For comparison, we also estimated the proportion of patients with access to “other lethal means” who received a safety intervention.

We reported the proportion who received a safety intervention overall and by specific patient characteristics. Among those who did not receive any safety intervention, we differentiated between those for whom a safety plan was not attempted and those who were offered but declined to complete one. Given that VA clinical guidance and memoranda require delivering safety interventions to patients who are discharged home and stratified as being at intermediate (acute or chronic) or high (acute or chronic) suicide risk,^14^,^20^ we also conducted a subgroup analysis to describe the proportion of patients who received safety interventions if their acute or chronic risk was intermediate or high. We used either chi-square tests or Fisher exact tests (if any expected cell counts were < 5) to assess for significant differences across groups for categorical outcomes. For continuous outcomes, we used either analysis of variance or independent sample *t*-tests. The sample sizes in all groups were substantially > 30 and, thus, met the assumptions for the methods used (ie, the central limit theorem is in effect). We used SAS v9.4 (SAS Institute Inc, Cary, NC) for all analyses.

## RESULTS

We identified 17,194 patients who sought care in VHA ED/UC settings, received a CSRE, and had a discharge disposition of “home.” Sociodemographic and clinical characteristics of the full population and by documented access to lethal means are shown in Table 1. Results of CSRE suicide risk stratification are shown in Table 2. Overall, 15.2% were documented as having firearm access, 8.5% as having access to “other” lethal means, 68.8% as having no lethal means access, and 7.4% as having unknown lethal means access (Table 1). The characteristics of the 2,378 patients who declined to receive a VA Safety Plan were generally similar to those of the overall population (Appendix Table 1).

In comparison to other patients, those documented as having firearm access were significantly more likely to be male, White, non-Hispanic, and married, and were less likely to have a mental health diagnosis or to have engaged VHA homelessness services (Table 1). A stratification of intermediate or high acute suicide risk was determined for 31.6% of those with firearm access; for 46.8% of those with access to “other” means; 20.6% of those with no lethal means access; and 29.1% of those with “unknown” lethal means access (*P* < .001). A stratification of intermediate or high chronic suicide risk was determined for 48.5% of those with firearm access, 70.0% of those with access to “other” means, 46.3% of those with no lethal means access, and 53.2% of those with unknown lethal means access (*P* < .001).

Of the 2,624 patients with documented firearm access, 80.6% were documented as having received a safety intervention within 24 hours of their ED encounter (Table 3). Of those, 56.8% received lethal means safety counseling, 13.2% received a firearm cable lock, and 88.6% reviewed or completed a new or updated VA Safety Plan. For comparison, of the 1,459 patients with documented access to “other” lethal means, 77.5% were documented as having received a safety intervention. Among those with documented firearm access, receipt of a safety intervention was significantly more likely among those who were younger, non-White, assessed as being at intermediate (acute or chronic) or high (acute or chronic) suicide risk rather than low (acute or chronic) risk, or had a positive C-SSRS Screener during the encounter (*P* < .01, all comparisons). Receipt of a safety intervention ranged from 68.0% among those at low acute, low chronic risk to 95.2% among those at intermediate acute, low chronic risk. In a subgroup analysis of 1,441 patients with documented firearm access who were assessed as having intermediate or high acute or chronic suicide risk, 90.8% were documented as having received a safety intervention (Appendix Table 2).

## DISCUSSION

The VHA has implemented a universal suicide risk screening and evaluation approach for patients receiving care in ED and UC settings. To our knowledge, this is the first study to describe patient-reported, clinician-documented access to firearms and other lethal means, as well as safety interventions delivered to those patients. We found that an overall low proportion of patients are identified as having firearm access (15%). However, among those with firearm access, 8 in 10 patients received a safety intervention within 24 hours of their discharge.

Nationally representative studies of the U.S. veteran population suggest that about one-half reside in a household with a firearm^17^; a substantially lower prevalence than among the national population of veterans who sought care in VA ED/UC settings and underwent suicide risk evaluation (among whom the true prevalence of firearm access is unknown). Some of this difference is likely attributable to sociodemographic differences between these populations. For example, the average income of U.S. firearm owners is higher than among non-owners.^21^ Nearly one-half of the patients in this study had previously used homeless services, suggesting that they may have lower-than-average income. Because we specifically identified a group of patients undergoing suicide risk evaluation, we may have further selected for a population with lower-than-average firearm ownership if some patients had taken steps to reduce their access to firearms prior to their encounter.^22^ Consistent with our findings, a recent evaluation of safety planning in the VHA found that only 28% of veterans were documented as having firearm access.^23^ Lower than expected prevalences of firearm access have also been identified within the context of mental health treatment in other healthcare systems.^24^

The difference in firearm access between these populations may also be partially attributable to screening and reporting practices. Prior studies have identified concerns among veterans and other firearm owners about disclosing firearm access during clinical encounters, particularly within the context of mental health problems.^25^–^29^ Similar studies have also identified barriers to asking about firearm access during encounters, including competing demands, lack of training, and discomfort with firearm-related discussions.^27^,^30^,^31^ Regarding the latter, however, the VHA has dedicated significant resources to training its healthcare workforce to engage in discussions about firearms, particularly among clinicians who would be expected to engage in suicide risk evaluation in ED/UC settings.

When patients undergoing suicide risk evaluation were identified as having firearm access, 81% received a safety intervention within 24 hours (91% among those with intermediate or high acute or chronic risk). This highlights room for improvement in terms of ensuring delivery of safety interventions for all patients with elevated suicide risk. Notably, we were unable to capture safety interventions that occurred but were not documented appropriately, and we did not assess delivery of interventions > 24 hours after the episode of care. If interventions are being delivered outside that 24-hour window, it might suggest that processes are in place to deliver recommended care, and further study would be needed to identify obstacles to doing so in a timelier fashion.

Among those with firearm access, receipt of safety interventions was lowest among those assessed as having low acute, low chronic risk for suicide (68%; notably, a VA Safety Plan is not required for these patients). The likelihood of receiving a safety intervention then increased as acute or chronic risk increased. Unexpectedly, patients who were assessed as having high acute risk for suicide were less likely to receive a safety intervention than most other risk groups. Such a finding is disconcerting as those at high acute risk are presumably designated as such in part due to their inability to maintain safety autonomously. Additional work is needed to ensure the validity of these initial risk stratifications and what clinical care such patients do receive. However, we also conducted exploratory post hoc analyses to further investigate this issue by identifying each of these patients and determining whether they received additional clinical care (yes/no) in the following 24 hours (rather than being discharged home without short-term treatment).

In those analyses, most patients appeared to be receiving intensive suicide-specific care around the time of their ED/UC encounter. Of the 81 patients who were stratified as having high acute risk for suicide, had access to a firearm, and who had a discharge disposition of “home,” 79 had an additional clinical encounter in the 24 hours after ED arrival and 39 had an additional clinical encounter in the 24 hours after ED departure. In many cases, clinicians coordinated same-day or short-term follow-up in mental health settings (during which safety interventions are often delivered). In other situations, patients were transferred to other care settings or hospitalized immediately after their ED/UC discharge. Notably, a discharge disposition of “home” does not necessarily mean that a patient returned directly to their home.

We identified several areas for further investigation regarding the use of the CSRE to document access to lethal means. First, 69% of patients were documented as having no access to lethal means. Given the ubiquity of potential lethal means, such as ligatures and sharp instruments, this estimate seems implausible. This may indicate that clinicians assessed patients’ access to means and felt that no specific lethal method was of particular relevance to the patient’s suicide risk. Further study of this issue among clinicians could inform modifications to the CSRE (eg, clarity around item intent). Second, further work is needed to understand why and under what conditions a clinician would document that a patient had “unknown” access to lethal means (eg, patient declined to answer; patient intoxicated), which accounted for 7% of this study population.

Nearly one-half of patients in this study population had previously accessed VHA homelessness services. This highlights the importance of developing safety interventions that are specific to the needs of patients with housing instability.^32^ For example, interventions that promote secure in-home firearm storage are less likely to be applicable to unsheltered homeless veterans. Given that most veterans keep firearms for personal and household protection, and that homeless patients are at increased risk of physical and sexual violence, identifying ways to increase the personal safety of these patients is likely to be a necessary step in addressing firearm access among those with elevated suicide risk.^33^,^34^

## LIMITATIONS

This study has limitations. First, we describe delivery of safety interventions among those with firearm access. While firearm-specific interventions are recommended for this population, we were unable to confirm with existing data whether these interventions included firearm content. Second, we considered endorsement of having completed a safety plan on the CSRE template as having received a safety intervention and did not specifically review VA Safety Plan templates themselves. However, in exploratory post hoc analyses, of those identified as having received a safety plan on the CSRE template, 95.5% had a documented safety plan note within 24 hours of their care episode. Third, although our approach to evaluating care delivery could be applied elsewhere, our specific findings are not generalizable to other settings and populations.

## CONCLUSION

The Veterans Health Administration has initiated a nationwide suicide risk screening and evaluation program. Among patients who underwent suicide risk evaluation through this program in ED or UC settings, 15% had patient-reported, clinician-documented firearm access and nearly 81% of those individuals received a safety intervention within 24 hours of being discharged home. These findings highlight the substantial reach and potential of such an initiative, as well as opportunities for process improvement.

## Supplementary Information





## Figures and Tables

**Table 1:** Demographic, clinical, and suicide risk characteristics of study population, by self-reported, clinician-documented access to lethal means.

	Total	Firearm access	“Other” lethal means access	No lethal means access	Unknown access	*P-*value
	N = 17,194	n = 2,624 (15.2%)	n = 1,459 (8.5%)	n = 11,832 (68.8%)	n = 1,279 (7.4%)	
Age, mean (SD)	50 (16.8)	50 (17.7)	49 (16.2)	50 (16.8)	51 (15.7)	.33
Age groups, n (%)						< .001
<18–39	5,714 (33.2)	961 (36.6)	508 (34.8)	3,878 (32.8)	367 (28.7)	
40–64	7,419 (43.1)	965 (36.8)	635 (43.5)	5,179 (43.8)	640 (50)	
65–79	3,528 (20.5)	594 (22.6)	279 (19.1)	2,421 (20.5)	234 (18.3)	
> 80	533 (3.1)	104 (4)	37 (2.5)	354 (3)	38 (3)	
Sex, n (%)						< .001
Male	14,834 (86.3)	2361 (90)	1,208 (82.8)	10,146 (85.8)	1,119 (87.5)	
Female	2,360 (13.7)	263 (10)	251 (17.2)	1,686 (14.2)	160 (12.5)	
Race, n (%)						< .001
White	9,794 (57)	1,654 (63)	906 (62.1)	6,523 (55.1)	711 (55.6)	
Black	4,788 (27.8)	599 (22.8)	321 (22)	3,516 (29.7)	352 (27.5)	
Unknown	1,974 (11.5)	296 (11.3)	167 (11.4)	1,337 (11.3)	174 (13.6)	
American Indian / Alaska Native	218 (1.3)	32 (1.2)	24 (1.6)	147 (1.2)	15 (1.2)	
Asian	227 (1.3)	26 (1)	24 (1.6)	164 (1.4)	13 (1)	
Native Hawaiian / Pacific Islander	193 (1.1)	17 (0.6)	17 (1.2)	145 (1.2)	14 (1.1)	
Ethnicity, n (%)						< .001
Not Hispanic / Latino	14,023 (81.6)	2213 (84.3)	1,171 (80.3)	9,598 (81.1)	1,041 (81.4)	
Hispanic / Latino	1,689 (9.8)	183 (7)	157 (10.8)	1,234 (10.4)	115 (9)	
Unknown	1,482 (8.6)	228 (8.7)	131 (9)	1,000 (8.5)	123 (9.6)	
Marital status, n (%)						< .001
Single	10,452 (60.8)	1,231 (46.9)	893 (61.2)	7,571 (64)	757 (59.2)	
Married	6,317 (36.7)	1324 (50.5)	530 (36.3)	3,981 (33.6)	482 (37.7)	
Unknown	425 (2.5)	69 (2.6)	36 (2.5)	280 (2.4)	40 (3.1)	
Mental health diagnosis, n (%)	15,451 (89.9)	2,266 (86.4)	1,371 (94)	10,650 (90)	1,164 (91)	< .001
Homeless services, n (%)	8,288 (48.2)	537 (20.5)	803 (55)	6,271 (53)	677 (52.9)	< .001
Acute Risk, n (%)						< .001
Low	12,872 (74.9)	1,795 (68.4)	776 (53.2)	9,394 (79.4)	907 (70.9)	
Intermediate	3,810 (22.2)	746 (28.4)	579 (39.7)	2,211 (18.7)	274 (21.4)	
High	506 (2.9)	81 (3.1)	103 (7.1)	225 (1.9)	97 (7.6)	
Chronic Risk, n (%)						< .001
Low	8,737 (50.8)	1,352 (51.5)	437 (30)	6,350 (53.7)	598 (46.8)	
Intermediate	7,452 (43.3)	1,139 (43.4)	846 (58)	4,894 (41.4)	573 (44.8)	
High	995 (5.8)	131 (5)	174 (11.9)	583 (4.9)	107 (8.4)	
Patient-level risk stratification, n (%)						< .001
Acute low, chronic low	8,003 (46.5)	1,181 (45)	353 (24.2)	5,910 (49.9)	559 (43.7)	
Acute low, chronic intermediate	4,538 (26.4)	584 (22.3)	381 (26.1)	3,249 (27.5)	324 (25.3)	
Acute low, chronic high	329 (1.9)	30 (1.1)	42 (2.9)	233 (2)	24 (1.9)	
Acute intermediate, chronic low	701 (4.1)	165 (6.3)	80 (5.5)	421 (3.6)	35 (2.7)	
Acute intermediate, chronic inter	2,691 (15.7)	516 (19.7)	414 (28.4)	1,551 (13.1)	210 (16.4)	
Acute intermediate, chronic high	416 (2.4)	65 (2.5)	84 (5.8)	238 (2)	29 (2.3)	
Acute high, chronic low	33 (0.2)	6 (0.2)	4 (0.3)	19 (0.2)	4 (0.3)	
Acute high, chronic intermediate	223 (1.3)	39 (1.5)	51 (3.5)	94 (0.8)	39 (3)	
Acute high, chronic high	250 (1.5)	36 (1.4)	48 (3.3)	112 (0.9)	54 (4.2)	
Positive C-SSRS Screen, n (%)*	7,783 (48.4)	1,264 (50.5)	884 (64.3)	5,063 (45.9)	572 (49.5)	< .001

* Estimate is the percentage of positive C-SSRS screens among those who received a C-SSRS assessment.

*SD*, standard deviation; *C-SSRS*, Columbia Suicide Severity Rating Scale.

**Table 2:** Acute and chronic suicide risk stratification of U.S. veterans population (N = 17,194)* in a study of patients who received care in Veterans Health Administration emergency departments or urgent care centers.

	Chronic risk, n (%)**				

		Low	Intermediate	High	Total
Acute risk, n (%)	Low	8,003 (46.5)	4,538 (26.4)	329 (1.9)	12,872 (74.9)
	Intermediate	701 (4.1)	2,691 (15.7)	416 (2.4)	3,810 (22.2)
	High	33 (0.2)	223 (1.3)	250 (1.5)	506 (2.9)
	Total	8,737 (50.8)	7,452 (43.3)	995 (5.8)	17,194

* Ten patients were missing risk stratification data.

** Percentages in each cell reflect the proportion of those patients within the entire population.

**Table 3:** Receipt of any safety intervention based on demographic, clinical, and suicide risk characteristics of patients with self-reported, clinician-documented firearm access (n = 2,624).

	Received safety intervention	Declined VA Safety Plan / No other safety intervention	No safety intervention	*P*-value

	n = 2,114 (80.6)	n = 127 (4.8)	n = 383 (14.6)	
Age, mean (SD)	49 (17.4)	54 (20.3)	53 (18.2)	< .001
Age groups, n (%)				< .001
<18–39	803 (83.6)	40 (4.2)	118 (12.3)	
40–64	785 (81.3)	40 (4.1)	140 (14.5)	
65–79	461 (77.6)	32 (5.4)	101 (17)	
80+	65 (62.5)	15 (14.4)	24 (23.1)	
Sex, n (%)				.06
Male	1,888 (80)	116 (4.9)	357 (15.1)	
Female	226 (85.9)	11 (4.2)	26 (9.9)	
Race, n (%)				< .001
White	1,289 (77.9)	95 (5.7)	270 (16.3)	
Black	515 (86)	15 (2.5)	69 (11.5)	
Unknown	246 (83.1)	15 (5.1)	35 (11.8)	
American Indian / Alaska Native	29 (90.6)	1 (3.1)	2 (6.3)	
Asian	21 (80.8)	1 (3.8)	4 (15.4)	
Native Hawaiian / Pacific Islander	14 (82.4)	0 (0)	3 (17.6)	
Ethnicity, n (%)				.27
Not Hispanic / Latino	1,768 (79.9)	110 (5)	335 (15.1)	
Hispanic / Latino	158 (86.3)	6 (3.3)	19 (10.4)	
Unknown	188 (82.5)	11 (4.8)	29 (12.7)	
Marital status, n (%)				.11
Single	989 (80.3)	65 (5.3)	177 (14.4)	
Married	1,063 (80.3)	58 (4.4)	203 (15.3)	
Unknown	62 (89.9)	4 (5.8)	3 (4.3)	
Mental health diagnosis, n (%)				.46
Yes	1,819 (80.3)	114 (5)	333 (14.7)	
No	295 (82.4)	13 (3.6)	50 (14)	
Homelessness, n (%)				.93
Yes	434 (80.8)	27 (5)	76 (14.2)	
No	1,680 (80.5)	100 (4.8)	307 (14.7)	
Acute risk, n (%)				<.001
Low	1,346 (75)	96 (5.3)	353 (19.7)	
Intermediate	701 (94)	24 (3.2)	21 (2.8)	
High	65 (80.2)	7 (8.6)	9 (11.1)	
Chronic Risk, n (%)				<.001
Low	965 (71.4)	62 (4.6)	325 (24)	
Intermediate	1,031 (90.5)	59 (5.2)	49 (4.3)	
High	116 (88.5)	6 (4.6)	9 (6.9)	
Patient-level risk stratification, n (%)				<.001
Low acute, low chronic	803 (68)	57 (4.8)	321 (27.2)	
Low acute, intermediate chronic	515 (88.2)	38 (6.5)	31 (5.3)	
Low acute, high chronic	28 (93.3)	1 (3.3)	1 (3.3)	
Intermediate acute, low chronic	157 (95.2)	5 (3)	3 (1.8)	
Intermediate acute, intermediate chronic	483 (93.6)	18 (3.5)	15 (2.9)	
Intermediate acute, high chronic	61 (93.8)	1 (1.5)	3 (4.6)	
High acute, low chronic	5 (83.3)	0 (0)	1 (16.7)	
High acute, intermediate chronic	33 (84.6)	3 (7.7)	3 (7.7)	
High acute, high chronic	27 (75)	4 (11.1)	5 (13.9)	
C-SSRS Screener, n (%)*				< .001
Positive	1,117 (88.4)	58 (4.6)	89 (7)	
Negative	900 (72.7)	60 (4.8)	278 (22.5)	

* Estimate is the percentage of positive C-SSRS screens among those who received a C-SSRS Screener.

*SD*, standard deviation; *C-SSRS*, Columbia Suicide Severity Rating Scale.
