# Supplementary material for: Clinician-documented Firearm Access and Safety Interventions for Veterans Receiving Suicide Risk Evaluation in VA Emergency Care Settings
Source: West J Emerg Med. 2026 May 19;27(3):784–93. doi: 10.5811/westjem.50852 (PMC13246209; doi:10.5811/westjem.50852)
Supplement: Supplementary file 1 [file wjem-27-784-s001.docx]

| **Appendix Table 1.** Demographic, clinical, and suicide risk characteristics of overall study population compared to 2,378 patients who declined to receive a Safety Plan | | |
| --- | --- | --- |
|  | Total | Declined Safety Plan |
|  | n=17194 | n=2378 |
| Age, mean (SD) | 50 (16.8) | 52 (16.3) |
| Age groups, n (%) |  |  |
| <18-39 | 5714 (33.2) | 658 (27.7) |
| 40-64 | 7419 (43.1) | 1102 (46.3) |
| 65-79 | 3528 (20.5) | 521 (21.9) |
| 80+ | 533 (3.1) | 97 (4.1) |
| Sex, n (%) |  |  |
| Male | 14834 (86.3) | 2075 (87.3) |
| Female | 2360 (13.7) | 303 (12.7) |
| Race, n (%) |  |  |
| White | 9794 (57) | 1343 (56.5) |
| Black / African American | 4788 (27.8) | 648 (27.2) |
| Unknown | 1974 (11.5) | 299 (12.6) |
| American Indian / Alaska Native | 227 (1.3) | 34 (1.4) |
| Asian | 218 (1.3) | 30 (1.3) |
| Native Hawaiian / Pacific Islander | 193 (1.1) | 24 (1) |
| Ethnicity, n (%) |  |  |
| Not Hispanic / Latino | 14023 (81.6) | 1957 (82.3) |
| Hispanic / Latino | 1689 (9.8) | 194 (8.2) |
| Unknown | 1482 (8.6) | 227 (9.5) |
| Marital status, n (%) |  |  |
| Single | 10452 (60.8) | 1560 (65.6) |
| Married | 6317 (36.7) | 729 (30.7) |
| Unknown | 425 (2.5) | 89 (3.7) |
| Mental health diagnosis, n (%) | 15451 (89.9) | 2159 (90.8) |
| Homeless services, n (%) | 8288 (48.2) | 1353 (56.9) |
| Acute Risk, n (%) |  |  |
| Low | 12872 (74.9) | 1778 (74.8) |
| Intermediate | 3810 (22.2) | 508 (21.4) |
| High | 506 (2.9) | 92 (3.9) |
| Chronic Risk, n (%) |  |  |
| Low | 8737 (50.8) | 1020 (42.9) |
| Intermediate | 7452 (43.3) | 1206 (50.7) |
| High | 995 (5.8) | 151 (6.3) |
| Risk Strata, n (%) |  |  |
| Low acute, low chronic | 8003 (46.5) | 918 (38.6) |
| Low acute, intermediate chronic | 4538 (26.4) | 813 (34.2) |
| Low acute, high chronic | 329 (1.9) | 47 (2) |
| Intermediate acute, low chronic | 701 (4.1) | 92 (3.9) |
| Intermediate acute, intermediate chronic | 2691 (15.7) | 359 (15.1) |
| Intermediate acute, high chronic | 416 (2.4) | 56 (2.4) |
| High acute, low chronic | 33 (0.2) | 10 (0.4) |
| High acute, intermediate chronic | 223 (1.3) | 34 (1.4) |
| High acute, high chronic | 250 (1.5) | 48 (2) |
| Positive C-SSRS Screener, n (%)* | 7783 (48.4) | 1203 (54.5) |
| *Estimate is the percentage of positive C-SSRS screens among those who received a C-SSRS Screener | | |

*SD*, standard deviation; *C-SSRS*, Columbia Suicide Severity Rating Scale
