# Supplementary material for: Clinician-documented Firearm Access and Safety Interventions for Veterans Receiving Suicide Risk Evaluation in VA Emergency Care Settings
Source: West J Emerg Med. 2026 May 19;27(3):784–93. doi: 10.5811/westjem.50852 (PMC13246209; doi:10.5811/westjem.50852)
Supplement: Supplementary file 2 [file wjem-27-784-s002.docx]

| **Appendix Table 2.** Receipt of any safety intervention based on demographic, clinical, and suicide risk characteristics of patients with self-reported, clinician-documented firearm access and intermediate or high acute or chronic risk (n=1,441) | | | |
| --- | --- | --- | --- |
|  | Received safety intervention | Declined Safety Plan / No safety intervention | No safety intervention |
|  | n=1309  (90.8) | n=70  (4.9) | n=62  (4.3) |
| Age, mean (SD) | 47 (17.0) | 53 (20.9) | 49 (17.8) |
| Age groups, n (%) |  |  |  |
| <18-39 | 541 (91.2) | 26 (4.4) | 26 (4.4) |
| 40-64 | 478 (92.1) | 20 (3.9) | 21 (4) |
| 65-79 | 263 (90.7) | 14 (4.8) | 13 (4.5) |
| 80+ | 27 (69.2) | 10 (25.6) | 2 (5.1) |
| Sex, n (%) |  |  |  |
| Male | 1145 (90.5) | 64 (5.1) | 56 (4.4) |
| Female | 164 (93.2) | 6 (3.4) | 6 (3.4) |
| Race, n (%) |  |  |  |
| White | 838 (89.7) | 58 (6.2) | 38 (4.1) |
| Black / African American | 276 (92.9) | 6 (2) | 15 (5.1) |
| Unknown | 150 (92) | 6 (3.7) | 7 (4.3) |
| American Indian / Alaska Native | 19 (95) | 0 (0) | 1 (5) |
| Asian | 16 (100) | 0 (0) | 0 (0) |
| Native Hawaiian / Pacific Islander | 10 (90.9) | 0 (0) | 1 (9.1) |
| Ethnicity, n (%) |  |  |  |
| Not Hispanic / Latino | 1094 (90.6) | 62 (5.1) | 52 (4.3) |
| Hispanic / Latino | 99 (92.5) | 3 (2.8) | 5 (4.7) |
| Unknown | 116 (92.1) | 5 (4) | 5 (4) |
| Marital status, n (%) |  |  |  |
| Single | 642 (89.9) | 41 (5.7) | 31 (4.3) |
| Married | 628 (91.4) | 28 (4.1) | 31 (4.5) |
| Unknown | 39 (97.5) | 1 (2.5) | 0 (0) |
| Mental health diagnosis, n (%) |  |  |  |
| Yes | 1141 (90.3) | 65 (5.1) | 57 (4.5) |
| No | 168 (94.4) | 5 (2.8) | 5 (2.8) |
| Homelessness, n (%) |  |  |  |
| Yes | 295 (88.9) | 17 (5.1) | 20 (6) |
| No | 1014 (91.4) | 53 (4.8) | 42 (3.8) |
| Acute Risk, n (%) |  |  |  |
| Low | 543 (88.4) | 39 (6.4) | 32 (5.2) |
| Intermediate | 701 (94) | 24 (3.2) | 21 (2.8) |
| High | 65 (80.2) | 7 (8.6) | 9 (11.1) |
| Chronic Risk, n (%) |  |  |  |
| Low | 162 (94.7) | 5 (2.9) | 4 (2.3) |
| Intermediate | 1031 (90.5) | 59 (5.2) | 49 (4.3) |
| High | 116 (88.5) | 6 (4.6) | 9 (6.9) |
| Patient-level risk stratification, n (%) |  |  |  |
| Low acute, low chronic | - | - | - |
| Low acute, intermediate chronic | 515 (88.2) | 38 (6.5) | 31 (5.3) |
| Low acute, high chronic | 28 (93.3) | 1 (3.3) | 1 (3.3) |
| Intermediate acute, low chronic | 157 (95.2) | 5 (3) | 3 (1.8) |
| Intermediate acute, intermediate chronic | 483 (93.6) | 18 (3.5) | 15 (2.9) |
| Intermediate acute, high chronic | 61 (93.8) | 1 (1.5) | 3 (4.6) |
| High acute, low chronic | 5 (83.3) | 0 (0) | 1 (16.7) |
| High acute, intermediate chronic | 33 (84.6) | 3 (7.7) | 3 (7.7) |
| High acute, high chronic | 27 (75) | 4 (11.1) | 5 (13.9) |
| C-SSRS Screener, n (%)* |  |  |  |
| Positive | 789 (92.4) | 37 (4.3) | 28 (3.3) |
| Negative | 454 (88.5) | 30 (5.8) | 29 (5.7) |
| *Estimate is the percentage of positive C-SSRS screens among those who received a C-SSRS Screener | | | |

*SD*, standard deviation; *C-SSRS*, Columbia Suicide Severity Rating Scale
